# Systemic dysregulation of essential and toxic elements and redox homeostasis in gynecologic malignancies

**DOI:** 10.3389/fonc.2026.1770641

**Published:** 2026-05-15

**Authors:** Paweł Ordon, Kacper Boroń, Krzysztof Bereza, Dariusz Boroń, Piotr Ossowski, Tomasz Sirek, Agata Sirek, Julia Gajdeczka, Beniamin Oskar Grabarek

**Affiliations:** 1 Collegium Medicum, WSB University, Dabrowa Gornicza, Poland; 2 Department of Plastic Surgery, Faculty of Medicine, Academy of Silesia, Katowice, Poland; 3 Department of Mother and Child Health, Faculty of Health Sciences, Institute of Nursing and Midwifery, Jagiellonian University Medical College, Cracow, Poland; 4 Faculty of Medicine and Health Sciences, Andrzej Frycz Modrzewski University in Kraków, Kraków, Poland; 5 Department of Gynecology and Obstetrics, TOMMED Specjalisci od Zdrowia, Katowice, Poland; 6 Department of Gynecology and Obstetrics with Gynecologic Oncology, Ludwik Rydygier Memorial Specialized Hospital, Kraków, Poland; 7 Department of Plastic and Reconstructive Surgery, Hospital for Minimally Invasive and Reconstructive Surgery in Bielsko-Biała, Bielsko-Biala, Poland

**Keywords:** endometrial cancer, ovarian cancer, oxidative stress, toxic metals, trace elements

## Abstract

**Background:**

Endometrial cancer (EC) and ovarian cancer (OC) are among the most prevalent gynecologic malignancies, yet systemic biochemical alterations associated with these diseases remain incompletely characterized. Disturbances in essential and toxic element homeostasis, together with redox imbalance, have been implicated in cancer-related metabolic changes. This study aimed to comprehensively evaluate circulating macro- and trace elements alongside oxidative stress markers in women with EC and OC.

**Methods:**

Serum and whole-blood concentrations of sodium (Na), potassium (K), calcium (Ca), phosphorus (P), magnesium (Mg), manganese (Mn), copper (Cu), zinc (Zn), lead (Pb), cadmium (Cd), and iron (Fe) were quantified using inductively coupled plasma optical emission spectrometry (ICP-OES). Systemic redox status was assessed by measuring total antioxidant status (TAS), total oxidant status (TOS), and calculating the oxidative stress index (OSI). Analyses were conducted in women with endometrial cancer stratified by tumor grade, patients with ovarian cancer stratified by treatment strategy, and corresponding control groups. Associations with age, body mass index, menopausal status, and type 2 diabetes were also evaluated.

**Results:**

In EC, higher tumor grade was associated with significantly lower circulating levels of Ca, Mg, and Fe, along with increased concentrations of Mn, Cu, Pb, and Cd. Decreasing trends were also observed for K and Zn. A comparable pattern was identified in OC, with the most pronounced alterations in patients requiring surgery followed by chemotherapy. Correlation analyses revealed significant inter-element relationships, particularly involving Mg, Cu, Pb, and Fe. Redox profiling demonstrated a progressive decrease in TAS and corresponding increases in TOS and OSI across tumor grades and clinical subgroups in both malignancies (p < 0.001 for all). Demographic and metabolic factors showed weak and inconsistent associations, with limited explanatory power of regression models.

**Conclusion:**

Endometrial and ovarian cancers are associated with distinct yet partially overlapping alterations in systemic elemental composition and redox balance. These changes are stage-associated and may reflect complex, non-specific interactions between disease, host response, and environmental factors, although causality cannot be established. Combined multi-element and redox profiling may provide complementary insights into the systemic biochemical characteristics of gynecologic malignancies; however, further longitudinal and mechanistic studies are required to clarify their clinical relevance.

## 1 Introduction

Endometrial cancer (EC) and ovarian cancer (OC) are among the most significant malignancies affecting women in the post-reproductive period (1–3). Although these cancers differ in their clinical presentation and progression, both are strongly influenced by hormonal changes, metabolic disturbances, and lifestyle factors (4–7). Endometrial cancer is frequently detected at an early stage and is closely associated with obesity, insulin resistance, and postmenopausal endocrine alterations (8, 9). In contrast, ovarian cancer is well known for its silent development and late diagnosis, which substantially reduces survival outcomes (3, 10, 11). Despite ongoing advances in diagnostic imaging and molecular profiling, there is a continued need for simple, reliable, blood-based biomarkers that could improve early detection, guide therapeutic decisions, or help monitor disease progression (12, 13).

In recent years, researchers have increasingly focused on the role of essential and toxic elements circulating in the bloodstream (14–17). Essential trace elements—such as zinc (Zn), copper (Cu), magnesium (Mg), iron (Fe), and manganese (Mn)—are critical for maintaining genomic stability, supporting antioxidant enzymes, regulating mitochondrial function, and modulating inflammatory responses (18–20). When the balance of these elements is disturbed, physiological processes can shift toward chronic inflammation, oxidative stress, and carcinogenesis (21–24). At the same time, toxic elements, including cadmium (Cd) and lead (Pb), can promote DNA damage, disrupt endocrine pathways, and interfere with normal cell repair mechanisms (25–28). Together, these alterations may leave a measurable imprint on systemic biochemistry that could reflect tumor presence or biological activity (29).

Oxidative stress is another key factor in the development and progression of gynecologic cancers (30). It arises when the production of reactive oxygen species exceeds the body’s ability to neutralize them, resulting in damage to DNA, lipids, and proteins (31). Elevated oxidative stress has been implicated in tumor initiation, aggressive behavior, and resistance to chemotherapy in both EC and OC (30, 32).

Importantly, many lifestyle and metabolic factors known to influence cancer risk—such as smoking, obesity, type 2 diabetes mellitus (T2DM), and menopausal status—also affect trace element concentrations and oxidative balance (33, 34). Endometrial cancer, for example, is strongly associated with excess body weight and insulin resistance (35), whereas ovarian cancer comprises biologically diverse subtypes that exhibit variable metabolic Characteristics (36).

Using inductively coupled plasma optical emission spectrometry (ICP-OES), we quantified a panel of major and trace elements in serum and whole blood (37). In parallel, we assessed Total Antioxidant Status (TAS), Total Oxidant Status (TOS), and Oxidative Stress Index (OSI) to characterize systemic redox balance (38). By examining these parameters across tumor grades, age categories, metabolic profiles, and clinical characteristics, we sought to identify element-based or redox-related patterns associated with gynecologic malignancies.

Based on this background, the present study aimed to perform a comprehensive analysis of circulating essential and toxic elements, along with oxidative stress markers, in women with endometrial cancer and ovarian cancer compared with women undergoing gynecologic surgery for benign conditions.

## 2 Materials and methods

### 2.1 Ethical considerations

All procedures were conducted in accordance with the ethical standards of the Declaration of Helsinki (2013 revision). The study protocol was approved by the Bioethical Committee of the Regional Medical Chamber in Kraków (approval No. 17/KBL/OIL/2024, dated 27 May 2025). Written informed consent was obtained from all participants prior to inclusion. To ensure confidentiality, all personal identifiers were removed before data processing, and anonymized data were used throughout the study.

### 2.2 Study population

#### 2.2.1 Endometrial cancer cohort

Seventy women scheduled for hysterectomy were enrolled in the study. The study group consisted of 50 patients with histologically confirmed endometrioid endometrial adenocarcinoma, while 20 women undergoing gynecological surgery for benign conditions served as controls.

All participants were over 45 years of age and post-reproductive. The diagnosis of endometrial cancer was established prior to enrollment based on histopathological examination of samples obtained during hysteroscopy-guided curettage. Following diagnosis, all patients underwent standard surgical treatment consisting of total hysterectomy with pelvic and para-aortic lymphadenectomy.

Exclusion criteria included endometriosis, adenomyosis, non-endometrioid carcinoma, adenocarcinoma with squamous differentiation, coexisting cervical cancer, hormone therapy within two years prior to surgery, morbid obesity (BMI > 40 kg/m²), and a history of other malignancies.

Tumor grading was performed according to standard criteria, with 28 cases classified as G1, 13 as G2, and 9 as G3. Detailed demographic and clinical characteristics are presented in **Table 1**.

**Table 1:** Baseline demographic and clinical characteristics of control subjects and patients with endometrial cancer.

Variable		C (n =20)	G1 (n =28)	G2 (n = 13)	G3 (n = 9)
Age	<50 years	3	6	1	0
	50–60 years	9	12	5	3
	>60 years	8	10	7	6
BMI [kg/m^2^]	Normal	8	6	2	1
	Overweight	7	11	4	3
	Obese (<40)	5	11	7	5
Cigarette smoking	Yes	6	9	5	4
	No	14	19	8	5
Menopause status	Yes	16	22	12	9
	No	4	6	1	0
Type 2 diabetes	Yes	3	7	5	4
	No	17	21	8	5

Data are presented as n. BMI, body mass index; C, control group; G1–G3, endometrial cancer histological grades 1–3.

#### 2.2.2 Ovarian cancer cohort

A total of 41 women with histologically confirmed ovarian cancer (FIGO stages I–IV) were included. Patients were stratified according to treatment approach.

Group A (n = 28) included patients undergoing surgical tumor resection followed by first-line adjuvant chemotherapy (8 stage I, 7 stage II, and 13 stage III cases). Group B (n = 13) consisted of patients with early-stage type I ovarian cancer treated surgically without adjuvant therapy. The control group (Group C; n = 25) included women undergoing hysterectomy with adnexectomy for benign conditions.

Detailed clinical and demographic data are summarized in **Table 2**.

**Table 2:** Baseline demographic and clinical characteristics of control subjects and patients with ovarian cancer.

Variable		Group A (surgery + chemotherapy; n = 28)	Group B (surgery only; n = 13)	Group C (n= 25)
Age	<50 years	3	4	5
	50–60 years	10	5	9
	>60 years	15	4	11
BMI [kg/m^2^]	Normal	6	5	9
	Overweight	11	4	8
	Obese (BMI <40)	11	4	8
Cigarette smoking	Yes	10	4	7
	No	18	9	18
Menopause status	Yes	23	10	18
	No	5	3	7
Type 2 diabetes	Yes	9	3	4
	No	19	10	21

Data are presented as n. BMI, body mass index; Group A, ovarian cancer patients treated with surgery plus chemotherapy; Group B, ovarian cancer patients treated with surgery only; Group C, control group.

### 2.3 Assessment of menopausal status, lifestyle variables, and metabolic comorbidities

Information on menopausal status, smoking habits, BMI, and type 2 diabetes mellitus (T2DM) was collected during preoperative evaluation.

Menopause was defined as the absence of menstruation for at least 12 consecutive months, excluding secondary causes. Smoking status was classified as current smoker or non-smoker based on a standardized questionnaire.

BMI (kg/m²) was calculated from measured height and weight and categorized as normal weight, overweight, or obese (BMI < 40 kg/m², in accordance with exclusion criteria).

T2DM diagnosis was confirmed based on medical history, pharmacological treatment, or laboratory criteria (HbA1c ≥ 6.5% and/or fasting plasma glucose ≥ 126 mg/dL).

### 2.4 Blood collection and sample handling

Venous blood samples were collected from each participant at hospital admission prior to surgical intervention. Two 5-mL samples were obtained from the antecubital vein using trace-element–free EDTA tubes (S-Monovette, Sarstedt, Germany).

One sample was centrifuged (3000 rpm, 15 min, room temperature; ~704 × g) to obtain serum. Both whole blood and serum aliquots were transferred into polypropylene tubes, coded, and stored at −80 °C until analysis.

All procedures were performed using metal-free equipment under a dedicated laminar flow hood to minimize contamination.

### 2.5 Preparation of samples for element quantification

Samples were thawed at room temperature and subjected to acid digestion. Aliquots of 0.75–1.0 mL of serum or whole blood were transferred into Teflon digestion vessels, followed by the addition of concentrated nitric acid (65% HNO_3_, Suprapur, Merck, Germany) and a 30-minute pre-digestion step under a fume hood. Subsequently, hydrogen peroxide (30% H_2_O_2_, Suprapur, Merck) was added to ensure complete oxidation of organic components.

Microwave-assisted digestion was performed using a MARS 5 system (CEM, USA), with a controlled temperature ramp to 180 °C over 15 minutes, followed by a 20-minute holding phase at 180 °C. After cooling, the digested samples were diluted according to the analytical requirements, with a 20-fold dilution applied for macroelements (Na, K, Ca, P, Mg) and a 5-fold dilution for trace elements (Mn, Cu, Zn, Pb, Cd, Fe).

All samples were spiked with yttrium at a final concentration of 0.5 mg/L as an internal standard and supplemented with 1% Triton X-100 to improve analytical stability. The final volume was adjusted to 10 mL using 0.075% HNO_3_. Blank samples were processed using the same digestion and dilution protocol.

### 2.6 Determination of element concentrations in patients with endometrioid endometrial cancer, ovarian cancer, and control group

Elemental concentrations were determined using inductively coupled plasma optical emission spectrometry (ICP-OES; Optima 7300 DV, PerkinElmer, USA), equipped with a cyclonic spray chamber and concentric nebulizer.

Measurements were performed in axial or radial mode depending on analytical sensitivity.

Selected analytical wavelengths included:

Ca (315.887 nm), Zn (206.200 nm), Cu (224.700 nm), Fe (259.94 nm), Na (589.592 nm), P (178.284 nm), Mg (280.270 nm), Cd (228.802 nm), Pb (220.353 nm), and Mn (257.610 nm).

Calibration was conducted using certified multielement standards (Merck, Germany; AccuStandard, USA). Calibration curves demonstrated excellent linearity (R² = 0.999–1.000).

Analytical accuracy was verified using certified reference material (NIST SRM 8414, Bovine Muscle). Internal standard recovery ranged from 92% to 102%.

All measurements were performed in triplicate, and mean values were used for analysis.

### 2.7 Assessment of redox status

Serum total antioxidant status (TAS) and total oxidant status (TOS) were determined using commercial spectrophotometric assay kits (Randox Laboratories, UK, and MyBioSource, USA), in accordance with the manufacturers’ instructions. TAS values were expressed as mmol/L Trolox equivalents, whereas TOS concentrations were reported as µmol/L hydrogen peroxide equivalents. The oxidative stress index (OSI) was calculated as the ratio of TOS to TAS multiplied by 100.

### 2.8 Statistical analysis

Statistical analyses were performed using StatPlus software (AnalystSoft, USA).

Normality was assessed using the Shapiro–Wilk test, and homogeneity of variance was evaluated with Levene’s test. Continuous variables are presented as mean ± standard deviation (SD).

Group comparisons across multiple categories (tumor grade, age, BMI) were performed using one-way ANOVA followed by Tukey’s *post hoc* test. Two-group comparisons were conducted using independent-samples t-tests.

Univariate linear regression analyses were used to assess associations between clinical variables (age, BMI, menopausal status, diabetes) and elemental concentrations. Multivariate regression models were subsequently constructed to identify independent predictors.

Correlation analyses were performed using Pearson’s correlation coefficient.

Statistical significance was set at p < 0.05. All tests were two-sided.

## 3 Results

### 3.1 Differences in concentration of selected elements in women with endometrioid endometrial cancer and control group

As shown in **Table 3**, significant differences between the control group and endometrial cancer subgroups were observed for Ca, Mg, Mn, Cu, Pb, Cd, and Fe. Serum Ca and Mg levels were significantly lower in G3 compared with both the control group and G1. In contrast, Mn and Cu concentrations were significantly higher in G2 and G3 than in controls. Pb showed the most pronounced elevation, with the highest values in G3 and significantly higher levels than in all other groups; additionally, G2 exceeded control values. Cd concentrations were also significantly increased in G2 and G3 compared with controls. Fe levels were significantly reduced in G2 and G3 relative to the control group. No statistically significant differences were found for Na, K, P, or Zn, although K and Zn demonstrated a decreasing tendency with increasing tumor grade.

**Table 3:** Serum element concentrations in control subjects and endometrial cancer subgroups.

Element (unit)	Control ^©^	G1	G2	G3	ANOVA p-value	*Post hoc* (Tukey)
Na (mmol/L)	139.8 ± 2.9	141.1 ± 2.7	142.4 ± 3.3	141.0 ± 3.6	0.104	NS
K (mmol/L)	4.31 ± 0.27	4.15 ± 0.28	4.10 ± 0.31	4.06 ± 0.33	0.100	NS (trend ↓)
Ca (mmol/L)	2.33 ± 0.10	2.32 ± 0.10	2.30 ± 0.09	2.20 ± 0.09	0.004	C > G3; G1 > G3
P (mmol/L)	1.12 ± 0.19	1.08 ± 0.18	1.07 ± 0.23	1.19 ± 0.30	0.480	NS
Mg (mmol/L)	0.92 ± 0.08	0.91 ± 0.08	0.89 ± 0.08	0.80 ± 0.07	0.003	C > G3; G1 > G3
Mn (µg/dL)	0.0077 ± 0.0018	0.0091 ± 0.0021	0.0097 ± 0.0027	0.0115 ± 0.0021	<0.001	G3 > C; G2 > C
Cu (µg/dL)	98.1 ± 9.2	110.8 ± 11.7	111.4 ± 11.1	121.4 ± 12.1	<0.001	G3 > C; G2 > C
Zn (µg/dL)	102.6 ± 19.1	97.3 ± 16.9	95.7 ± 17.6	91.6 ± 21.8	0.459	NS
Pb (µg/dL)	1.86 ± 0.69	2.29 ± 0.85	2.47 ± 1.27	4.03 ± 0.88	<0.001	G3 > all; G2 > C
Cd (µg/L)	0.40 ± 0.13	0.42 ± 0.23	0.58 ± 0.17	0.65 ± 0.13	0.001	G3 > C; G2 > C
Fe (µmol/L)	22.7 ± 2.3	20.9 ± 3.6	20.6 ± 2.8	16.5 ± 4.0	<0.001	C > G3; C > G2

Data are presented as mean ± SD. Group comparisons were performed using one-way ANOVA followed by Tukey’s *post hoc* test. NS, not significant; C, control group; Na, sodium; K, potassium; Ca, calcium; P, phosphorus; Mg, magnesium; Mn, manganese; Cu, copper; Zn, zinc; Pb, lead; Cd, cadmium; Fe, iron.

According to **Table 4**, age had a limited influence on serum element concentrations. A significant difference was observed only for Na, with the 50–60-year group differing from both <50 and >60 years. No significant age-related differences were found for the remaining elements. However, Cd showed a tendency to increase with age, with a *post hoc* difference between <50 and >60 years, while Fe demonstrated a borderline decrease in the oldest group.

**Table 4:** Serum element concentrations according to age in the endometrial cancer cohort.

Element	<50 yrs	50–60 yrs	>60 yrs	p (ANOVA)	*Post hoc* (Tukey)
Na (mmol/L)	141.14 ± 2.67	142.42 ± 3.26	141.02 ± 3.62	0.0115*	<50 vs 50–60; 50–60 vs >60
K (mmol/L)	4.15 ± 0.28	4.10 ± 0.31	4.06 ± 0.33	0.8503	–
Ca (mmol/L)	2.32 ± 0.10	2.30 ± 0.09	2.20 ± 0.09	0.3772	–
P (mmol/L)	1.08 ± 0.18	1.07 ± 0.23	1.19 ± 0.30	0.7041	–
Mg (mmol/L)	0.91 ± 0.08	0.89 ± 0.08	0.80 ± 0.07	0.6241	–
Mn (µg/dL)	0.00911 ± 0.00213	0.00969 ± 0.00274	0.01145 ± 0.00206	0.7136	–
Cu (µg/dL)	110.85 ± 11.72	111.44 ± 11.06	121.41 ± 12.09	0.3634	–
Zn (µg/dL)	97.25 ± 16.88	95.65 ± 17.56	91.56 ± 21.83	0.5046	–
Pb (µg/dL)	2.29 ± 0.85	2.47 ± 1.27	4.03 ± 0.88	0.4870	–
Cd (µg/L)	0.42 ± 0.23	0.58 ± 0.17	0.65 ± 0.13	0.1226	–
Fe (µmol/L)	20.88 ± 3.62	20.62 ± 2.79	16.50 ± 3.96	0.0586	–

Data are presented as mean ± SD. Comparisons between age categories were performed using one-way ANOVA followed by Tukey’s *post hoc* test. Na, sodium; K, potassium; Ca, calcium; P, phosphorus; Mg, magnesium; Mn, manganese; Cu, copper; Zn, zinc; Pb, lead; Cd, cadmium; Fe, iron.

As presented in **Table 5**, BMI was not associated with significant differences in most elements. The only exception was Mn, which was significantly higher in overweight patients compared with those with normal BMI. All other elements, including toxic metals, remained comparable across BMI categories.

**Table 5:** Serum element concentrations according to BMI category in the endometrial cancer cohort.

Element	Normal	Overweight	Obese <40	P (ANOVA)	*Post hoc* (Tukey)
Na (mmol/L)	140.74 ± 3.61	140.74 ± 2.53	141.32 ± 3.19	0.7439	NS
K (mmol/L)	4.25 ± 0.28	4.22 ± 0.29	4.09 ± 0.31	0.1513	NS
Ca (mmol/L)	2.29 ± 0.10	2.32 ± 0.09	2.30 ± 0.12	0.8057	NS
P (mmol/L)	1.09 ± 0.16	1.10 ± 0.21	1.12 ± 0.24	0.9045	NS
Mg (mmol/L)	0.92 ± 0.10	0.88 ± 0.08	0.90 ± 0.08	0.4237	NS
Mn (µg/dL)	0.0078 ± 0.0016	0.0097 ± 0.0021	0.0095 ± 0.0025	0.0302	Normal vs Overweight (p=0.036)
Cu (µg/dL)	104.85 ± 11.89	112.36 ± 10.14	107.72 ± 15.54	0.1685	NS
Zn (µg/dL)	96.02 ± 15.70	99.60 ± 20.38	97.15 ± 18.18	0.8076	NS
Pb (µg/dL)	2.10 ± 1.01	2.40 ± 1.02	2.63 ± 1.21	0.2984	NS
Cd (µg/L)	0.44 ± 0.23	0.51 ± 0.23	0.47 ± 0.16	0.5627	NS
Fe (µmol/L)	21.88 ± 3.53	20.04 ± 3.09	20.83 ± 4.08	0.2767	NS

Data are presented as mean ± SD. Comparisons between BMI categories were performed using one-way ANOVA followed by Tukey’s *post hoc* test. NS, not significant. BMI, body mass index; Na, sodium; K, potassium; Ca, calcium; P, phosphorus; Mg, magnesium; Mn, manganese; Cu, copper; Zn, zinc; Pb, lead; Cd, cadmium; Fe, iron.

Data shown in **Table 6** indicate that menopausal status did not significantly affect serum concentrations of any analyzed elements. Minor variations were observed but did not reach statistical significance.

**Table 6:** Serum element concentrations according to menopause status in the endometrial cancer cohort.

Element	Postmenopausal (mean ± SD)	Premenopausal (mean ± SD)	p (t-test)
Na (mmol/L)	141.61 ± 3.19	140.52 ± 1.18	0.112
K (mmol/L)	4.12 ± 0.30	4.13 ± 0.27	0.928
Ca (mmol/L)	2.29 ± 0.10	2.34 ± 0.14	0.338
P (mmol/L)	1.10 ± 0.23	1.06 ± 0.16	0.565
Mg (mmol/L)	0.89 ± 0.09	0.90 ± 0.07	0.606
Mn (µg/dL)	0.0098 ± 0.0025	0.0091 ± 0.0012	0.311
Cu (µg/dL)	113.40 ± 12.46	109.82 ± 9.59	0.402
Zn (µg/dL)	95.99 ± 17.58	94.72 ± 20.13	0.879
Pb (µg/dL)	2.58 ± 1.21	3.07 ± 0.69	0.154
Cd (µg/L)	0.52 ± 0.22	0.42 ± 0.18	0.225
Fe (µmol/L)	19.71 ± 3.81	21.96 ± 3.40	0.147

Data are presented as mean ± SD. Comparisons between postmenopausal and premenopausal patients were performed using Student’s t-test. Na, sodium; K, potassium; Ca, calcium; P, phosphorus; Mg, magnesium; Mn, manganese; Cu, copper; Zn, zinc; Pb, lead; Cd, cadmium; Fe, iron.

Similarly, **Table 7** demonstrates that the presence of type 2 diabetes had no significant impact on Na, K, Ca, P, Mg, Mn, Cu, Zn, Pb, Cd, or Fe levels.

**Table 7:** Serum element concentrations according to type 2 diabetes status in the endometrial cancer cohort.

Element (unit)	Diabetes (+)	Diabetes (–)	P-value (t-test)
Na (mmol/L)	140.82 ± 3.01	141.02 ± 2.86	0.71
K (mmol/L)	4.16 ± 0.34	4.18 ± 0.32	0.81
Ca (mmol/L)	2.31 ± 0.11	2.30 ± 0.10	0.69
P (mmol/L)	1.11 ± 0.24	1.10 ± 0.22	0.92
Mg (mmol/L)	0.89 ± 0.09	0.90 ± 0.09	0.69
Mn (µg/dL)	0.0090 ± 0.0026	0.0092 ± 0.0025	0.65
Cu (µg/dL)	111.2 ± 13.9	107.6 ± 12.8	0.41
Zn (µg/dL)	94.6 ± 19.8	99.1 ± 18.5	0.42
Pb (µg/dL)	2.56 ± 1.11	2.38 ± 1.03	0.67
Cd (µg/L)	0.50 ± 0.22	0.47 ± 0.20	0.72
Fe (µmol/L)	19.98 ± 4.02	21.12 ± 3.61	0.29

Data are presented as mean ± SD. Comparisons between patients with and without type 2 diabetes were performed using Student’s t-test. Na, sodium; K, potassium; Ca, calcium; P, phosphorus; Mg, magnesium; Mn, manganese; Cu, copper; Zn, zinc; Pb, lead; Cd, cadmium; Fe, iron.

The inter-element relationships presented in **Table 8** revealed several significant correlations. Mg was negatively correlated with Mn, Cu, and Pb, and positively correlated with Fe. Cu showed a positive correlation with Pb and a negative correlation with Fe. Additionally, Na was inversely correlated with Zn. These associations indicate coordinated systemic relationships between selected elements.

**Table 8:** Inter-element Pearson correlation matrix in the endometrial cancer cohort.

Variable	Na_	K_	Ca_	P_	Mg_	Mn_	Cu	Zn_	Pb_	Cd	Fe_
Na	1.000***	0.057	0.213	-0.035	0.032	0.166	-0.147	-0.323*	0.045	0.135	0.135
K_	0.057	1.000***	0.142	0.181	-0.125	-0.059	0.061	-0.006	-0.001	0.018	0.053
Ca_	0.213	0.142	1.000***	-0.213	0.178	-0.172	-0.194	-0.084	-0.119	-0.129	0.164
P	-0.035	0.181	-0.213	1.000***	-0.229	0.009	0.128	-0.094	0.148	-0.076	0.001
Mg	0.032	-0.125	0.178	-0.229	1.000***	-0.341*	-0.461***	0.114	-0.279*	-0.247	0.315*
Mn	0.166	-0.059	-0.172	0.009	-0.341*	1.000***	0.258	0.125	0.217	-0.036	-0.201
Cu	-0.147	0.061	-0.194	0.128	-0.461***	0.258	1.000***	-0.026	0.281*	0.153	-0.335*
Zn	-0.323*	-0.006	-0.084	-0.094	0.114	0.125	-0.026	1.000***	-0.192	0.021	0.078
Pb_	0.045	-0.001	-0.119	0.148	-0.279*	0.217	0.281*	-0.192	1.000***	0.126	-0.263
Cd	0.135	0.018	-0.129	-0.076	-0.247	-0.036	0.153	0.021	0.126	1.000***	-0.111
Fe_	0.135	0.053	0.164	0.001	0.315*	-0.201	-0.335*	0.078	-0.263	-0.111	1.000***

r, Pearson correlation coefficients are shown. Statistical significance was defined as * p < 0.05, ** p < 0.01, and *** p < 0.001. Na, sodium; K, potassium; Ca, calcium; P, phosphorus; Mg, magnesium; Mn, manganese; Cu, copper; Zn, zinc; Pb, lead; Cd, cadmium; Fe, iron.

In the univariate analysis summarized in **Table 9**, tumor grade was positively associated with Mn, Cu, Pb, and Cd, and negatively associated with Ca, Mg, and Fe. Age showed a positive association with Fe and inverse associations with Pb and Cd, while other clinical variables demonstrated weak relationships.

**Table 9:** Univariate regression analysis of clinical factors associated with serum element concentrations in the endometrial cancer cohort.

Element	Grade β	Age β	BMI β	Menopause β	Diabetes β
Na	+0.04	–0.04	–0.06	+0.13	–0.00
K	–0.11	+0.00	+0.22	–0.01	–0.03
Ca	–0.44	–0.01	+0.01	–0.19	+0.12
P	+0.16	–0.24	–0.03	+0.07	–0.05
Mg	–0.44	+0.09	+0.06	–0.07	+0.08
Mn	+0.35	–0.18	–0.17	+0.09	–0.12
Cu	+0.29	–0.10	–0.01	+0.10	+0.01
Zn	–0.12	–0.04	–0.08	+0.03	–0.02
Pb	+0.51	–0.22	–0.08	–0.15	–0.07
Cd	+0.42	–0.21	+0.05	+0.16	+0.01
Fe	–0.38	+0.28	–0.01	–0.21	–0.08

Results are presented as β coefficients from univariate regression analyses. BMI, body mass index; Na, sodium; K, potassium; Ca, calcium; P, phosphorus; Mg, magnesium; Mn, manganese; Cu, copper; Zn, zinc; Pb, lead; Cd, cadmium; Fe, iron.

As shown in **Table 10**, multivariate analysis confirmed tumor grade as an independent predictor of lower Ca, Mg, and Fe levels and higher Pb and Cd concentrations. Smoking was additionally associated with lower Mg levels. However, the R² values were low to moderate, indicating limited explanatory power of the models.

**Table 10:** Multivariate regression analysis of serum element concentrations in the endometrial cancer cohort.

Element	Grade B (95% CI)	Age B (95% CI)	BMI B (95% CI)	Menopause B (95% CI)	Diabetes B (95% CI)	Smoking B (95% CI)	R²
Na	–0.037 (–1.304 to 1.230)	–0.227 (–1.892 to 1.438)	–0.253 (–1.676 to 1.170)	+0.873 (–2.403 to 4.148)	–0.067 (–2.248 to 2.114)	+0.639 (–1.259 to 2.537)	0.034
K	–0.043 (–0.162 to 0.076)	+0.010 (–0.155 to 0.175)	+0.094 (–0.051 to 0.239)	–0.015 (–0.348 to 0.318)	–0.026 (–0.248 to 0.195)	+0.130 (–0.049 to 0.308)	0.109
Ca	–0.064 (–0.102 to –0.026)	–0.002 (–0.054 to 0.050)	+0.003 (–0.042 to 0.049)	–0.079 (–0.183 to 0.026)	+0.050 (–0.020 to 0.120)	+0.001 (–0.056 to 0.058)	0.266
P	+0.028 (–0.061 to 0.118)	–0.078 (–0.200 to 0.044)	–0.009 (–0.117 to 0.099)	+0.024 (–0.224 to 0.272)	–0.018 (–0.183 to 0.147)	+0.057 (–0.078 to 0.191)	0.090
Mg	–0.049 (–0.080 to –0.018)	+0.019 (–0.023 to 0.061)	+0.015 (–0.022 to 0.052)	–0.031 (–0.115 to 0.054)	+0.013 (–0.044 to 0.070)	–0.049 (–0.096 to –0.003)	0.292
Mn	+0.001 (0.000 to 0.002)	–0.001 (–0.003 to 0.001)	–0.001 (–0.003 to 0.001)	+0.000 (–0.004 to 0.004)	–0.001 (–0.004 to 0.002)	–0.000 (–0.002 to 0.001)	0.179
Cu	+4.576 (–0.369 to 9.520)	–1.525 (–7.929 to 4.880)	–0.118 (–5.580 to 5.344)	+1.595 (–11.002 to 14.193)	+0.203 (–8.208 to 8.614)	–1.017 (–8.427 to 6.392)	0.090
Zn	–3.138 (–10.513 to 4.236)	–0.140 (–9.746 to 9.466)	–1.092 (–9.264 to 7.080)	+0.967 (–17.792 to 19.726)	–0.733 (–13.274 to 11.808)	–6.706 (–17.756 to 4.345)	0.063
Pb	+0.863 (0.470 to 1.255)	–0.218 (–0.725 to 0.290)	–0.077 (–0.510 to 0.356)	–0.186 (–1.181 to 0.808)	–0.089 (–0.754 to 0.575)	–0.123 (–0.711 to 0.465)	0.382
Cd	+0.117 (0.033 to 0.201)	–0.059 (–0.167 to 0.049)	+0.017 (–0.076 to 0.110)	+0.058 (–0.156 to 0.272)	+0.003 (–0.140 to 0.146)	–0.008 (–0.134 to 0.118)	0.208
Fe	–1.501 (–2.944 to –0.057)	+1.010 (–0.863 to 2.883)	–0.020 (–1.610 to 1.571)	–0.820 (–4.475 to 2.835)	–0.283 (–2.720 to 2.154)	–1.338 (–3.501 to 0.825)	0.220

Results are presented as B coefficients with 95% CI, confidence intervals from multivariate regression analyses adjusted for grade, age, BMI, menopause status, diabetes, and smoking. R², coefficient of determination; BMI, body mass index; Na, sodium; K, potassium; Ca, calcium; P, phosphorus; Mg, magnesium; Mn, manganese; Cu, copper; Zn, zinc; Pb, lead; Cd, cadmium; Fe, iron.

### 3.2 Differences in concentration of selected elements in women with ovarian cancer and control group

As shown in **Table 11**, significant differences between ovarian cancer groups and controls were observed for most analyzed elements. Na levels were higher in both Group A and Group B compared with controls. In contrast, K was significantly lower in Group A relative to the control group. Ca concentrations were reduced in Group A compared with both Group B and controls, while P levels were significantly elevated in Group A compared with the remaining groups. Mg levels were lower in Group A than in controls. Mn and Cu showed consistent increases across groups, with the highest values in Group A, followed by Group B and controls, and all pairwise differences reaching statistical significance. Pb and Cd concentrations were also elevated, with the highest levels in Group A; Cd additionally differed significantly between Group B and controls. No significant differences were found for Zn. Fe showed a non-significant trend toward lower levels in Group B compared with controls.

**Table 11:** Serum element concentrations in ovarian cancer groups and controls.

Element	Group A (mean ± SD)	Group B (mean ± SD)	Group C (mean ± SD)	ANOVA p-value	*Post hoc* (significant comparisons)
Na (mmol/L)	141.99 ± 1.86	141.19 ± 1.54	139.83 ± 2.03	0.0004	A > C, B > C
K (mmol/L)	4.06 ± 0.25	4.21 ± 0.29	4.27 ± 0.30	0.0218	A < C
Ca (mmol/L)	2.26 ± 0.07	2.31 ± 0.08	2.36 ± 0.08	0	A < B, A < C
P (mmol/L)	1.20 ± 0.14	1.09 ± 0.13	1.04 ± 0.13	0.0004	A > B, A > C
Mg (mmol/L)	0.87 ± 0.06	0.89 ± 0.07	0.91 ± 0.07	0.0331	A < C
Mn (µg/dL)	0.01 ± 0.00	0.01 ± 0.00	0.01 ± 0.00	0	A > B, A > C, B > C
Cu (µg/dL)	118.40 ± 8.88	110.42 ± 10.30	97.35 ± 11.46	0	A > B, A > C, B > C
Zn (µg/dL)	99.17 ± 17.94	95.28 ± 13.34	102.41 ± 9.06	0.3408	NS
Pb (µg/dL)	3.07 ± 0.74	2.36 ± 0.66	1.91 ± 0.88	0	A > B, A > C
Cd (µg/L)	0.56 ± 0.07	0.43 ± 0.11	0.35 ± 0.09	0	A > B, A > C, B > C
Fe (µmol/L)	19.68 ± 3.92	19.24 ± 2.55	21.72 ± 3.79	0.0662	B < C

Data are presented as mean ± SD. Group comparisons were performed using one-way ANOVA followed by Tukey’s *post hoc* test. NS, not significant; Group A, ovarian cancer patients treated with surgery plus chemotherapy; Group B, ovarian cancer patients treated with surgery only; Group C, control group; Na, sodium; K, potassium; Ca, calcium; P, phosphorus; Mg, magnesium; Mn, manganese; Cu, copper; Zn, zinc; Pb, lead; Cd, cadmium; Fe, iron.

According to **Table 12**, age was not associated with significant differences in any of the analyzed elements. All parameters, including both essential and toxic elements, remained comparable across age groups.

**Table 12:** Serum element concentrations according to age in the ovarian cancer cohort.

Element	<50 yrs (mean ± SD)	50–60 yrs (mean ± SD)	>60 yrs (mean ± SD)	P (ANOVA)	Tukey
Na (mmol/L)	140.37 ± 2.52	141.20 ± 2.17	141.14 ± 1.78	0.484	NS
K (mmol/L)	4.22 ± 0.30	4.10 ± 0.27	4.17 ± 0.28	0.482	NS
Ca (mmol/L)	2.31 ± 0.08	2.30 ± 0.07	2.31 ± 0.07	0.513	NS
P (mmol/L)	1.12 ± 0.14	1.11 ± 0.13	1.12 ± 0.13	0.744	NS
Mg (mmol/L)	0.89 ± 0.07	0.87 ± 0.07	0.91 ± 0.07	0.589	NS
Mn (µg/dL)	0.009 ± 0.003	0.010 ± 0.003	0.009 ± 0.003	0.801	NS
Cu (µg/dL)	107.62 ± 13.52	106.56 ± 11.87	111.13 ± 12.46	0.915	NS
Zn (µg/dL)	97.61 ± 16.78	102.42 ± 13.67	98.22 ± 13.61	0.180	NS
Pb (µg/dL)	2.41 ± 0.78	2.43 ± 0.78	2.57 ± 0.86	0.292	NS
Cd (µg/L)	0.44 ± 0.12	0.45 ± 0.15	0.46 ± 0.12	0.387	NS
Fe (µmol/L)	20.46 ± 2.85	20.62 ± 3.00	19.87 ± 4.14	0.726	NS

Data are presented as mean ± SD. Comparisons between age categories were performed using one-way ANOVA followed by Tukey’s *post hoc* test. NS, not significant. Na, sodium; K, potassium; Ca, calcium; P, phosphorus; Mg, magnesium; Mn, manganese; Cu, copper; Zn, zinc; Pb, lead; Cd, cadmium; Fe, iron.

As presented in **Table 13**, BMI had a limited impact on elemental concentrations. A significant difference was observed only for Mg, which was lower in overweight individuals compared with obese patients. No other elements showed significant variation across BMI categories.

**Table 13:** Serum element concentrations according to BMI category in the ovarian cancer cohort.

Element	Normal (mean ± SD)	Overweight (mean ± SD)	Obese (mean ± SD)	P (ANOVA)	Tukey
Na (mmol/L)	141.13 ± 2.01	141.29 ± 2.01	140.65 ± 2.19	0.552	NS
K (mmol/L)	4.14 ± 0.28	4.10 ± 0.27	4.23 ± 0.29	0.344	NS
Ca (mmol/L)	2.31 ± 0.08	2.29 ± 0.07	2.33 ± 0.08	0.204	NS
P (mmol/L)	1.11 ± 0.13	1.11 ± 0.13	1.13 ± 0.14	0.134	NS
Mg (mmol/L)	0.90 ± 0.07	0.86 ± 0.07	0.91 ± 0.07	0.0101	Overweight < Obese<40 (p=0.003)
Mn (µg/dL)	0.009 ± 0.003	0.010 ± 0.003	0.010 ± 0.003	0.911	NS
Cu (µg/dL)	107.62 ± 13.52	106.56 ± 11.87	111.13 ± 12.46	0.971	NS
Zn (µg/dL)	97.61 ± 16.78	102.42 ± 13.67	98.22 ± 13.61	0.743	NS
Pb (µg/dL)	2.41 ± 0.78	2.43 ± 0.78	2.57 ± 0.86	0.440	NS
Cd (µg/L)	0.44 ± 0.14	0.47 ± 0.14	0.45 ± 0.11	0.665	NS
Fe (µmol/L)	20.41 ± 2.86	19.65 ± 3.20	20.72 ± 4.30	0.573	NS

Data are presented as mean ± SD. Comparisons between BMI categories were performed using one-way ANOVA followed by Tukey’s *post hoc* test. NS, not significant. BMI, body mass index; Na, sodium; K, potassium; Ca, calcium; P, phosphorus; Mg, magnesium; Mn, manganese; Cu, copper; Zn, zinc; Pb, lead; Cd, cadmium; Fe, iron.

Data shown in **Table 14** indicate that menopausal status did not significantly influence serum concentrations of any analyzed elements. Although K showed a borderline difference, it did not reach statistical significance.

**Table 14:** Serum element concentrations according to menopause status in the ovarian cancer cohort.

Element	Postmenopausal (mean ± SD)	Premenopausal (mean ± SD)	P (t-test)
Na (mmol/L)	141.12 ± 1.73	140.67 ± 2.99	0.586
K (mmol/L)	4.12 ± 0.29	4.29 ± 0.28	0.056
Ca (mmol/L)	2.29 ± 0.09	2.30 ± 0.07	0.672
P (mmol/L)	1.12 ± 0.14	1.09 ± 0.12	0.457
Mg (mmol/L)	0.88 ± 0.08	0.90 ± 0.08	0.371
Mn (µg/dL)	0.010 ± 0.001	0.010 ± 0.001	0.558
Cu (µg/dL)	108.60 ± 14.71	104.60 ± 9.34	0.216
Zn (µg/dL)	100.97 ± 14.36	100.02 ± 14.87	0.827
Pb (µg/dL)	2.50 ± 0.98	2.44 ± 1.21	0.856
Cd (µg/L)	0.48 ± 0.13	0.44 ± 0.14	0.411
Fe (µmol/L)	20.11 ± 3.71	20.75 ± 2.77	0.469

Data are presented as mean ± SD. Comparisons between postmenopausal and premenopausal patients were performed using Student’s t-test. Na, sodium; K, potassium; Ca, calcium; P, phosphorus; Mg, magnesium; Mn, manganese; Cu, copper; Zn, zinc; Pb, lead; Cd, cadmium; Fe, iron.

Similarly, **Table 15** demonstrates that the presence of type 2 diabetes had no significant effect on Na, K, Ca, P, Mg, Mn, Cu, Zn, Pb, Cd, or Fe levels.

**Table 15:** Serum element concentrations according to type 2 diabetes status in the ovarian cancer cohort.

Element	Diabetes (+) (mean ± SD)	Diabetes (–) (mean ± SD)	P (t-test)
Na (mmol/L)	141.36 ± 2.44	140.91 ± 1.94	0.512
K (mmol/L)	4.08 ± 0.30	4.18 ± 0.29	0.266
Ca (mmol/L)	2.30 ± 0.10	2.30 ± 0.08	0.847
P (mmol/L)	1.13 ± 0.15	1.11 ± 0.13	0.650
Mg (mmol/L)	0.91 ± 0.10	0.88 ± 0.07	0.340
Mn (µg/dL)	0.010 ± 0.001	0.010 ± 0.001	0.886
Cu (µg/dL)	108.91 ± 10.13	107.30 ± 14.75	0.626
Zn (µg/dL)	96.21 ± 17.22	102.21 ± 13.19	0.215
Pb (µg/dL)	2.43 ± 1.15	2.50 ± 1.00	0.830
Cd (µg/L)	0.50 ± 0.12	0.46 ± 0.13	0.238
Fe (µmol/L)	20.06 ± 3.66	20.31 ± 3.50	0.812

Data are presented as mean ± SD. Comparisons between patients with and without type 2 diabetes were performed using Student’s t-test. Na, sodium; K, potassium; Ca, calcium; P, phosphorus; Mg, magnesium; Mn, manganese; Cu, copper; Zn, zinc; Pb, lead; Cd, cadmium; Fe, iron.

The inter-element relationships presented in **Table 16** revealed several significant correlations. Na was positively correlated with Pb. K showed a negative correlation with Cu. Ca was inversely associated with Pb, while Mg was negatively correlated with Cd. Cu demonstrated a positive correlation with Cd. Additionally, Zn was negatively correlated with Fe. These findings indicate the presence of coordinated systemic associations between selected elements.

**Table 16:** Inter-element Pearson correlation matrix in the ovarian cancer cohort.

Variable	Na	K	Ca	P	Mg	Mn	Cu	Zn	Pb	Cd	Fe
Na	1.000***	0.108	-0.111	0.150	0.080	0.153	-0.177	0.137	0.373*	-0.189	-0.252
K	0.108	1.000***	-0.113	-0.146	-0.300	-0.170	-0.461**	-0.100	-0.000	-0.206	-0.225
Ca	-0.111	-0.113	1.000***	0.028	0.206	-0.077	-0.087	-0.096	-0.335*	0.067	0.021
P	0.150	-0.146	0.028	1.000***	0.006	-0.031	0.010	0.127	-0.155	0.075	-0.146
Mg	0.080	-0.300	0.206	0.006	1.000***	0.255	-0.038	0.194	0.028	-0.323*	0.071
Mn	0.153	-0.170	-0.077	-0.031	0.255	1.000***	0.257	-0.052	0.002	0.049	0.253
Cu	-0.177	-0.461**	-0.087	0.010	-0.038	0.257	1.000***	0.131	0.202	0.367*	-0.028
Zn	0.137	-0.100	-0.096	0.127	0.194	-0.052	0.131	1.000***	0.287	-0.063	-0.353*
Pb	0.373*	-0.000	-0.335*	-0.155	0.028	0.002	0.202	0.287	1.000***	-0.049	-0.108
Cd	-0.189	-0.206	0.067	0.075	-0.323*	0.049	0.367*	-0.063	-0.049	1.000***	-0.040
Fe	-0.252	-0.225	0.021	-0.146	0.071	0.253	-0.028	-0.353*	-0.108	-0.040	1.000***

r, Pearson correlation coefficients are shown. Statistical significance was defined as *p < 0.05, **p < 0.01, and ***p < 0.001. Na, sodium; K, potassium; Ca, calcium; P, phosphorus; Mg, magnesium; Mn, manganese; Cu, copper; Zn, zinc; Pb, lead; Cd, cadmium; Fe, iron.

In the univariate analysis summarized in **Table 17**, age showed positive associations with Na and Cu and a negative association with Fe. BMI was negatively associated with Na and Zn and positively associated with Fe. Menopause was positively associated with Na and Cu and negatively associated with Fe. Diabetes showed a negative association with Zn and Fe and a positive association with Na and Cu.

**Table 17:** Univariate regression analysis of clinical predictors of serum element concentrations in the ovarian cancer cohort.

Element	Age β	BMI β	Menopause β	Diabetes β
Na	+0.77	–0.34	+0.70	+0.48
K	–0.05	+0.08	–0.23	–0.11
Ca	–0.03	–0.02	+0.003	+0.003
P	+0.03	+0.02	+0.02	+0.02
Mg	–0.01	–0.002	–0.02	+0.02
Mn	~0	~0	+0.0002	~0
Cu	+4.5	–1.19	+4.91	+1.99
Zn	–2.1	–2.12	+1.45	–6.13
Pb	+0.13	–0.06	+0.09	–0.07
Cd	+0.02	~0	+0.04	+0.05
Fe	–0.59	+0.30	–0.86	–0.30

Results are presented as β coefficients from univariate regression analyses. BMI, body mass index; Na, sodium; K, potassium; Ca, calcium; P, phosphorus; Mg, magnesium; Mn, manganese; Cu, copper; Zn, zinc; Pb, lead; Cd, cadmium; Fe, iron.

As shown in **Table 18**, multivariate analysis did not identify any statistically significant independent predictors of elemental concentrations, as all confidence intervals included zero. The R² values were low, indicating limited explanatory power of the models.

**Table 18:** Multivariate regression analysis of serum element concentrations in the ovarian cancer cohort.

Element	Age B (95% CI)	BMI B (95% CI)	Menopause B (95% CI)	Diabetes B (95% CI)	R²
Na	+0.77 (–0.92 to 2.47)	–0.34 (–1.40 to 0.72)	+0.70 (–0.85 to 2.25)	+0.48 (–1.02 to 1.98)	0.05
K	–0.05 (–0.30 to 0.20)	+0.08 (–0.15 to 0.31)	–0.23 (–0.47 to 0.01)	–0.11 (–0.34 to 0.12)	0.06
Ca	–0.03 (–0.10 to 0.04)	–0.02 (–0.07 to 0.03)	+0.003 (–0.06 to 0.07)	+0.003 (–0.06 to 0.07)	0.04
P	+0.03 (–0.09 to 0.15)	+0.02 (–0.07 to 0.11)	+0.02 (–0.08 to 0.12)	+0.02 (–0.08 to 0.12)	0.05
Mg	–0.01 (–0.08 to 0.06)	–0.002 (–0.05 to 0.05)	–0.02 (–0.08 to 0.04)	+0.02 (–0.04 to 0.08)	0.04
Mn	~0 (–0.001 to 0.001)	~0 (–0.001 to 0.001)	+0.0002 (–0.001 to 0.001)	~0 (–0.001 to 0.001)	0.03
Cu	+4.5 (–6.2 to 15.2)	–1.19 (–5.8 to 3.4)	+4.91 (–3.9 to 13.7)	+1.99 (–5.2 to 9.2)	0.06
Zn	–2.1 (–10.4 to 6.2)	–2.12 (–7.9 to 3.7)	+1.45 (–6.2 to 9.1)	–6.13 (–14.3 to 2.0)	0.03
Pb	+0.13 (–0.80 to 1.06)	–0.06 (–0.58 to 0.46)	+0.09 (–0.70 to 0.88)	–0.07 (–0.80 to 0.66)	0.04
Cd	+0.02 (–0.12 to 0.16)	~0 (–0.09 to 0.09)	+0.04 (–0.08 to 0.16)	+0.05 (–0.07 to 0.17)	0.05
Fe	–0.59 (–3.2 to 2.0)	+0.30 (–1.2 to 1.8)	–0.86 (–3.6 to 1.9)	–0.30 (–2.8 to 2.2)	0.03

Results are presented as B coefficients with 95% confidence intervals (CI) from multivariate regression analyses adjusted for age, BMI, menopause status, and diabetes. R², coefficient of determination; BMI, body mass index; Na, sodium; K, potassium; Ca, calcium; P, phosphorus; Mg, magnesium; Mn, manganese; Cu, copper; Zn, zinc; Pb, lead; Cd, cadmium; Fe, iron.

### 3.3 Systemic redox imbalance in endometrial and ovarian cancer: comparative analysis of TAS, TOS, and OSI

Significant differences were observed for TAS, TOS, and OSI across groups (p<0.001 for all). TAS values decreased progressively from the control group to G1, G2, and G3, with significant differences between all compared groups. In contrast, TOS and OSI values increased across successive grades, with significant differences between the control group and all cancer grades, as well as between each pair of cancer grades. Detailed data are presented in **Table 19**.

**Table 19:** Serum TAS, TOS, and OSI in control subjects and endometrial cancer subgroups.

Variable	Control	G1	G2	G3	ANOVA p-value	Significant *Post Hoc* Differences
TAS (mmol/L)	1.62 ± 0.07	1.49 ± 0.04	1.34 ± 0.04	1.20 ± 0.03	<0.001	Control > G1, Control > G2, Control > G3; G1 > G2, G1 > G3; G2 > G3
TOS (µmol/L)	7.78 ± 0.93	10.23 ± 0.56	13.45 ± 0.66	16.13 ± 0.82	<0.001	Control < G1, Control < G2, Control < G3; G1 < G2, G1 < G3; G2 < G3
OSI	4.86 ± 0.79	6.86 ± 0.53	10.06 ± 0.72	13.48 ± 0.97	<0.001	Control < G1, Control < G2, Control < G3; G1 < G2, G1 < G3; G2 < G3

Data are presented as mean ± SD. Group comparisons were performed using one-way ANOVA followed by Tukey’s *post hoc* test. TAS, total antioxidant status; TOS, total oxidant status; OSI, oxidative stress index.

Significant differences were also observed for TAS, TOS, and OSI across ovarian cancer groups (p<0.001 for all). TAS values were lowest in group A, intermediate in group B, and highest in the control group, with significant differences between all groups. TOS and OSI showed the opposite pattern, with the highest values in group A, followed by group B and the control group, and significant differences between all groups. Detailed data are presented in **Table 20**.

**Table 20:** Serum TAS, TOS, and OSI in ovarian cancer groups and controls.

Variable	Group A (n=28)	Group B (n=13)	Group C (Control, n=25)	ANOVA p-value	Significant *post hoc* differences
TAS (mmol/L)	1.25 ± 0.14	1.33 ± 0.08	1.59 ± 0.15	<0.001	A < B, A < C, B < C
TOS (µmol/L)	14.76 ± 3.27	11.08 ± 2.75	8.42 ± 2.11	<0.001	A > B, A > C, B > C
OSI	12.01 ± 3.05	8.35 ± 2.07	5.35 ± 1.41	<0.001	A > B, A > C, B > C

Data are presented as mean ± SD. Group comparisons were performed using one-way ANOVA followed by Tukey’s *post hoc* test. Group A, ovarian cancer patients treated with surgery plus chemotherapy; Group B, ovarian cancer patients treated with surgery only; Group C, control group. TAS, total antioxidant status; TOS, total oxidant status; OSI, oxidative stress index.

## 4 Discussion

The elemental disturbances identified in this study reflect alterations in systemic metabolic and redox homeostasis associated with endometrial and ovarian malignancies. Importantly, these observations are based exclusively on circulating measurements (serum and whole blood), and therefore represent systemic biochemical signatures rather than direct assessments of intratumoral elemental composition. While the identified patterns are consistent with processes implicated in tumor biology, the present data do not allow causal inference and should be interpreted as indirect, systemic correlates of disease.

Although serum Na remained stable across disease categories, its systemic homeostasis does not preclude biologically relevant alterations at the cellular level. Na–H and Na–Ca exchangers regulate intracellular pH and calcium flux—processes frequently altered in proliferating, glycolytically active tumor cells. Experimental studies have demonstrated that changes in Na-dependent transport mechanisms may enhance cellular adaptation to acidic tumor microenvironments (39–41). However, given that the present study assessed only circulating Na levels, these interpretations remain speculative and cannot be directly linked to tumor-specific processes.

A downward trend in circulating K was observed with increasing malignancy, although this did not reach statistical significance. Experimental data suggest that extracellular K concentrations may influence immune signaling and apoptotic pathways (42), and dysregulated K channels have been implicated in tumor progression (43, 44). In the present study, reduced circulating K may reflect systemic alterations associated with malignancy; however, no direct mechanistic conclusions can be drawn from serum measurements alone (45–47).

Similarly, lower circulating Ca levels may reflect systemic redistribution or altered metabolic handling rather than direct tumor-specific depletion. Calcium signaling plays a central role in proliferation, apoptosis, mitochondrial function, and epithelial–mesenchymal transition (48, 49). Alterations in Ca transport pathways have been linked to cancer progression and therapy resistance (50–52).

Nevertheless, because only systemic concentrations were assessed, these findings should be interpreted as indirect indicators of altered calcium homeostasis.

No significant differences were observed for P, although its role in cellular metabolism remains well established. Phosphate is essential for nucleotide synthesis, ATP turnover, and membrane biosynthesis, and altered phosphate metabolism has been associated with cancer-related metabolic reprogramming (53). The absence of significant systemic differences suggests that circulating P may be tightly regulated despite potential intracellular alterations (54).

The increase in Mn may be associated with systemic responses to oxidative stress, as Mn serves as a cofactor for mitochondrial superoxide dismutase (SOD2), an enzyme frequently upregulated in cancer (55, 56). Elevated Mn levels observed in circulation may therefore reflect adaptive responses to increased oxidative burden rather than direct evidence of tumor-driven accumulation (57–59).

Among trace elements, elevated circulating Cu has been consistently reported in patients with gynecologic malignancies (29, 60). Copper is involved in angiogenesis, mitochondrial respiration, and extracellular matrix remodeling (61, 62). The observed increase in circulating Cu may therefore reflect systemic metabolic adaptations associated with tumor progression; however, given the lack of speciation data and tissue-level measurements, these findings should not be interpreted as direct evidence of Cu-mediated oncogenic mechanisms.

Although Zn levels did not differ significantly, a decreasing trend was observed. Zinc is essential for DNA repair, antioxidant defense, and structural stability of key regulatory proteins (63, 64). Reduced circulating Zn may reflect impaired systemic homeostasis or redistribution; however, its functional implications remain uncertain in the absence of intracellular or protein-bound fraction analysis.

Lower Fe levels demonstrated a pattern consistent with systemic metabolic remodeling in cancer. Inflammatory processes may promote iron sequestration, while tumor cells may increase iron uptake via transferrin receptors (65–67). Reduced circulating Fe may therefore reflect redistribution rather than absolute deficiency, consistent with mechanisms underlying anemia of chronic disease (68).

Elevated Pb and Cd levels may reflect environmental exposure and/or systemic redistribution processes associated with disease. Both metals are known to interfere with DNA repair, disrupt calcium signaling, and contribute to oxidative stress (69, 70).

However, an important limitation of this study is the lack of detailed exposure assessment and the inability to distinguish between pre-existing toxic burden and tumor-associated redistribution. Therefore, increased circulating Pb and Cd should be interpreted as associative findings rather than causal factors in carcinogenesis (71, 72). The observed correlations between elements suggest the presence of coordinated systemic alterations; however, these relationships should be interpreted with caution. Pearson correlations do not imply functional interactions, shared transport mechanisms, or regulatory coupling. The identified associations may reflect common systemic processes such as inflammation, oxidative stress, or altered protein binding, but remain hypothesis-generating and require validation in mechanistic studies (23, 27, 73). The pronounced redox imbalance observed in both malignancies is consistent with previous reports linking oxidative stress to cancer biology (74, 75). Reduced TAS and increased TOS and OSI indicate a shift toward a systemic pro-oxidative state. Reactive oxygen species are known to contribute to genomic instability and tumor progression (76). However, it should be emphasized that ICP-OES quantifies total elemental concentrations and does not distinguish between biologically active (free) and protein-bound metal fractions, which are critical determinants of redox activity. Therefore, the relationship between circulating metal levels and oxidative stress should be interpreted cautiously (77, 78).

Taken together, the findings of this study indicate that endometrial and ovarian cancers are associated with stage-related but non-linear alterations in systemic elemental profiles and redox balance. These patterns are likely associated with complex, bidirectional interactions between tumor biology, host metabolic response, and environmental influences, rather than direct causal mechanisms. Circulating elemental profiles may therefore serve as indirect biomarkers of disease-associated systemic dysregulation.

Several limitations of this study warrant careful consideration. First, the cross-sectional design precludes any inference regarding temporal relationships or causality, making it impossible to determine whether the observed elemental and redox alterations precede malignancy or arise as a consequence of tumor progression.

Second, the analysis is based exclusively on systemic measurements (serum and whole blood), which should be regarded as indirect surrogate markers. These measures do not reflect intratumoral elemental distribution, metal compartmentalization, or microenvironmental dynamics, and no correlation with tissue-level concentrations was performed.

Third, the study lacks comprehensive assessment of key confounding factors that may substantially influence elemental status, including environmental and occupational exposure, dietary intake, renal function, inflammatory status, and socioeconomic variables. In particular, the absence of structured exposure data limits the ability to distinguish between pre-existing toxic burden and disease-associated alterations.

Fourth, ICP-OES quantifies total elemental concentrations but does not provide information on metal speciation, oxidation state, or the distribution between free and protein-bound fractions, which are critical determinants of biological activity and redox potential.

Fifth, the statistical models demonstrated relatively low coefficients of determination, indicating limited explanatory power. Combined with modest subgroup sizes, particularly in higher-grade disease categories, this increases the risk of type I error, unstable estimates, and potential overfitting in multivariate analyses.

Finally, the use of correlation-based analyses does not allow inference of functional interactions, regulatory mechanisms, or shared transport pathways, and therefore the identified inter-element relationships should be interpreted as exploratory and hypothesis-generating.

Future studies should adopt longitudinal designs, incorporate detailed exposure assessment, and integrate systemic measurements with tissue-level analyses. Additional investigation of metal speciation, metalloproteins (e.g., metallothioneins), and transporter systems (e.g., CTR1, DMT1, ZIP/ZnT families) may further clarify the biological and clinical relevance of these findings. Therefore, the present findings should not be interpreted as evidence of direct causal or mechanistic relationships between elemental alterations and tumor biology.

## 5 Conclusion

Endometrial and ovarian cancers are associated with distinct yet partially overlapping alterations in systemic elemental composition and redox balance. Both malignancies are characterized by lower circulating levels of Fe, Ca, and Mg, accompanied by increasing concentrations of Mn, Cu, Pb, and Cd, while Zn and K showed non-significant decreasing trends. These patterns appear to be stage-associated rather than strictly linear, suggesting complex and dynamic changes in systemic homeostasis during disease progression.

Redox profiling further demonstrated a shift toward a pro-oxidative state, reflected by reduced TAS and increased TOS and OSI across malignant subgroups. Together, these findings indicate coordinated disturbances in systemic biochemical and oxidative parameters.

The relatively limited influence of the analyzed clinical variables, combined with low explanatory power of the regression models, suggests that these alterations may reflect multifactorial processes involving tumor biology, host metabolic response, and environmental influences, rather than direct or isolated effects of any single factor.

Overall, combined multi-element and redox profiling may provide complementary, exploratory insights into the systemic characteristics of gynecologic malignancies. However, given the cross-sectional design and the use of circulating surrogate markers, these findings should be interpreted with caution. Future studies incorporating longitudinal designs, detailed exposure assessment, tissue-level analyses, and clinical outcome data are required to determine the biological and clinical relevance of these observations.

## Data Availability

The original contributions presented in the study are included in the article/supplementary material. Further inquiries can be directed to the corresponding author.
